# Natural Drugs as a Treatment Strategy for Cardiovascular Disease through the Regulation of Oxidative Stress

**DOI:** 10.1155/2020/5430407

**Published:** 2020-09-27

**Authors:** Xing Chang, Tian Zhang, Wenjin Zhang, Zhenyu Zhao, Jiahui Sun

**Affiliations:** ^1^National Resource Center for Chinese Materia Medica, State Key Laboratory Breeding Base of Dao-di Herbs, China Academy of Chinese Medical Sciences, Beijing 100700, China; ^2^Guang'anmen Hospital, Chinese Academy of Traditional Chinese Medicine, Beijing 100053, China; ^3^Shandong University of Traditional Chinese Medicine, Jinan, Shandong 250355, China

## Abstract

Oxidative stress (OS) refers to the physiological imbalance between oxidative and antioxidative processes leading to increased oxidation, which then results in the inflammatory infiltration of neutrophils, increased protease secretion, and the production of a large number of oxidative intermediates. Oxidative stress is considered an important factor in the pathogenesis of cardiovascular disease (CVD). At present, active components of Chinese herbal medicines (CHMs) have been widely used for the treatment of CVD, including coronary heart disease and hypertension. Since the discovery of artemisinin for the treatment of malaria by Nobel laureate Youyou Tu, the therapeutic effects of active components of CHM on various diseases have been widely investigated by the medical community. It has been found that various active CHM components can regulate oxidative stress and the circulatory system, including ginsenoside, astragaloside, and resveratrol. This paper reviews advances in the use of active CHM components that modulate oxidative stress, suggesting potential drugs for the treatment of various CVDs.

## 1. Introduction

According to the World Health Organization (WHO) statistics, cardiovascular diseases (CVDs) were responsible for the highest number of deaths in 2019 [1]. Increasing and aging populations further complicate the situation, and 22.2 million CVD-related deaths are expected to occur in 2030 [2]. There is an important link between complex oxidation reactions and the development of atherosclerosis [3, 4]. Increasing levels of oxidative stress contribute to the subsequent formation and progression of atherosclerotic plaques [5]. A lack of endogenous antioxidants is another important cause of coronary heart disease [6]. The use of traditional medicinal plants has rapidly expanded in recent years. Medical plant research is no longer limited to chemical composition and pharmacology and now encompasses the study of metabolomics and underlying mechanisms of action [7]. Studies employ approaches ranging from simple chemical component separation and drug efficacy tests to transcriptomics and pathway research. The underlying mechanisms of the remarkable curative effects of medicinal plants are being revealed. Technological advances have greatly contributed to the increased use of medicinal plants. 25% of drugs in the global medical market are phytomedicines [8]. Further, 60% of anticancer drugs and 75% of drugs for the treatment of infectious diseases originate from medicinal plants [9]. Between 1984 and 2014, 9.1% of FDA-approved drugs were medicinal plants, while 21% were natural product derivatives [10].

Finding safe and effective drugs derived from natural products is a hot topic in the CVD field [11]. Medicinal plants have great advantages for the treatment of cardiovascular disease owing to their safety profiles [12]. Favorable effects of medicinal plants have been described for diseases such as hypertension, hyperlipidemia, atherosclerosis, and chronic heart failure, as well as for the overall reduction of cardiovascular risk [13]. Cardiovascular disease patients being informed about the benefits of medicinal plants could improve their health. Medicinal plant-containing diets may be used to effectively control hypertension, for example [14]. The sufficient intake of fruits, vegetables, nuts, red wine, coffee, and others can also effectively prevent the occurrence of CVD [15]. This is due to such foods often having antioxidant biological activities. Similarly, medicinal plants possess antioxidant pharmacological effects, making them likely drug candidates. Within medicinal plant research for CVD, oxidative stress inhibition is a very advanced research line. Accumulating evidence suggests that flavonoids, phenolics, and saponin from medical plants could reduce oxidative stress [16]. Our studies have evaluated the active ingredients of 20 different natural compounds, including ginsenoside, resveratrol, astragaloside A, and quercetin. The therapeutic mechanisms of these different active ingredients were addressed in the context of CVD. Herein, we provide an overview of the oxidative stress reduction mechanisms of medicinal plants for the treatment of CVD. The questions addressed include the following: (1) which medicinal plant components have antioxidative pharmacological effects? (2) What is the regulatory role of antioxidant medicinal plant components in CVD? (3) What is the mechanism of action of antioxidant medicinal plant components in CVD?

## 2. Methodology and Strategy

In this review, we performed a literature search for information on the bioactive components of medicinal plants and their effect on oxidative stress. The Institute for Scientific Information (ISI) Web of Knowledge, MEDLINE, PubMed, Scopus, Google Scholar, and the China National Knowledge Infrastructure (CNKI) databases were searched using relevant keywords and phrases, including “natural drugs and oxidative stress”, “natural active ingredients and oxidative stress”, “traditional Chinese medicine and oxidative stress”, “phytochemistry/medicinal plant extracts and oxidative stress”, “medicinal plants and cardiovascular diseases”, and “natural active ingredients and cardiovascular diseases”. From the search results, we manually selected original articles discussing natural medicines or their active ingredients and their effect(s) on oxidative stress or CVDs. Our search selection criteria were mainly based on the following:

*(1) Different Mechanisms Used by Natural Medicines and Active Ingredients to Treat Cardiovascular Diseases.* For example, total flavonoids of matsuba can dilate the coronary artery, increase blood flow, and reduce abnormal electron transfer of the myocardial cell membrane and the production of oxygen free radicals, thereby improving myocardial ischemia and treating coronary heart disease [17]. Orientin can protect red blood cells from oxidative damage by reducing oxidative stress, increasing the activity of antioxidant enzymes, and maintaining the structural integrity of red blood cells [18].

*(2) Different Categories of Natural Medicines and Active Ingredients with Therapeutic Efficacy in Cardiovascular Diseases.* The active ingredients of natural medicines including flavonoids, saponins, polyphenols, polysaccharides, and anthraquinones can exert different therapeutic effects. For example, saponin-based active ingredients have multiple functions such as antiviral activity, scavenging oxygen free radicals, expanding blood vessels, strengthening the heart, and reducing the synthesis of reactive oxygen species (ROS) and malondialdehyde (MDA) [19]. Polyphenols inhibit the formation of hydroxyl free radicals, increase endogenous superoxide dismutase (SOD) activity, inhibit lipid peroxidation, and increase cellular energy metabolism [20].

*(3) Different Therapeutic Targets of Natural Medicines and Active Ingredients.* For example, berberine can regulate adenosine 5′-monophosphate- (AMP-) activated protein kinase (AMPK) and mammalian target of rapamycin (mTOR) signaling pathways [21]. Curcumin can inhibit phosphatidylinositol 3-kinase- (PI3K-) serine/threonine protein kinase- (AKT-) mTOR signal transduction [22]. Further, some active ingredients can regulate multiple signal pathways and can also regulate the growth, proliferation, differentiation, migration, and apoptosis of a variety of cells.

## 3. Oxidative Stress and Cardiovascular Disease

Oxidative stress refers to the pathological state of reactive oxygen species (ROS) accumulation caused by the excessive production of oxygen radicals or a compromised intracellular antioxidant defense system [23]. Oxidative stress plays an important role in the regulation of the cardiovascular system and has become a new target for CVD prevention and treatment. Oxidative stress can cause severe functional damage to endothelial cells and cardiomyocytes [24, 25]. In addition, oxidative stress is involved in the pathogenesis of hypertension, myocardial ischemia-reperfusion injury, atherosclerosis, and other associated diseases by regulating inflammation and stimulating vascular smooth muscle proliferation [26–28].

**Figure 1:**
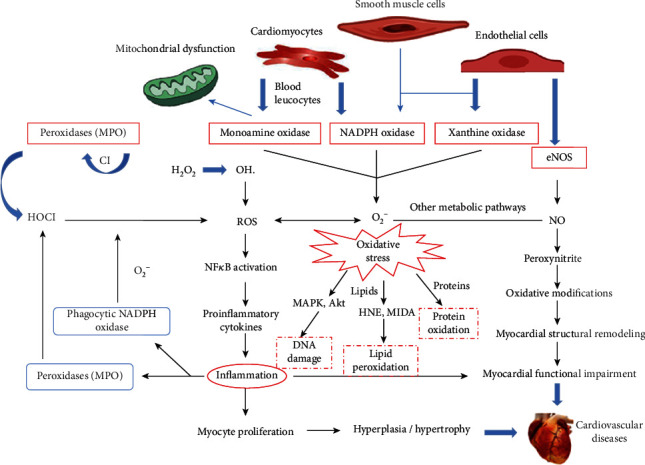
The factors that cause cardiovascular disease are complex, including NADPH, NOX, eNOS, NO, and ROS. ROS is the most obviously biological markers of oxidative stress. The increase in oxidative stress directly causes lipid peroxidation, protein and enzyme denaturation, nucleic acid DNA damage, and other mechanisms of the myocardial cell membrane or cardiovascular epithelial cells. These are also important pathways for the action of antioxidant drugs.

As shown in Figure 1, there are various biological markers of oxidative stress, among which ROS are the most closely linked to oxidative stress. In the normal physiological state of the human body, relatively small levels of endogenous ROS are produced, and, at a certain level, ROS play an important role in the protection of myocardial cells [29, 30]. However, in a pathological state, as ROS are scavenged at a rate that is much lower than the rate of their production, ROS will accumulate, directly leading to oxidative stress. Increased oxidative stress causes lipid peroxidation, protein and enzyme denaturation, DNA damage, and other events damaging the myocardial cell membrane or cardiovascular epithelial cells [31]. Further, oxidative stress and inflammation will cause myocardial injury and remodeling, leading to the occurrence and aggravation of CVD.

## 4. Application of Antioxidant Natural Drugs for Cardiovascular Disease

In order to reduce oxidative damage in cardiovascular tissue, an increasing number of medicinal plants are used as natural antioxidants in the clinic [32]. Active components with antioxidant activity can be isolated from various medicinal plants [33, 34]. Examples include phenols, flavonoids, and polysaccharides from traditional Chinese herbal medicine. As shown in Table 1, natural antioxidant drugs can inhibit the production of free radicals by enhancing specific and nonspecific immune function or by directly preventing free radical-induced cellular and tissue damage. The modern clinical treatment of CVD includes the use of natural drugs. Free radicals contain unpaired electrons, which have a tendency to pair. In the process of pairing, free radicals will generate more radicals, forming a chain reaction. Active components of traditional Chinese medicine can terminate this chain reaction both directly and indirectly. Further, these components can also regulate oxidative stress by reducing lipid peroxidation and free radical production, enhancing the scavenging of radicals, improving the activity of antioxidant enzymes, and upregulating the anti-inflammatory response [35]. Compared to synthetic antioxidants, natural medicinal plants with antioxidant properties have lower toxicity and thus a more favorable safety profile. Through these advantages, natural antioxidant medicinal plants may be used for targeting oxidative stress in the clinical treatment of CVD in the future.

### 4.1. Coronary Atherosclerotic Heart Disease

Coronary atherosclerotic heart disease (CHD) is a CVD caused by stenosis or obstruction of the coronary artery [36, 37]. It is characterized by the formation of lipid-filled atherosclerotic plaques under the tunica intima of the great and middle arteries, leading to stenosis or occlusion of the arterial lumen, which then causes myocardial ischemia and hypoxia [38, 39]. Various factors that lead to CHD may cause lipid peroxidation damage, promoting the occurrence and development of atherosclerosis [40, 41].

During early atherosclerosis, nutrients cannot pass freely due to the reduced blood flow, which in turn leads to a decrease in endogenous ATP levels, activates AMPK, inhibits mTOR, and causes increased ROS, leading to oxidative stress. Excessive ROS can cause endothelial and smooth muscle cell dysfunction. It can also lead to the activation of inflammatory signaling and cardiomyocyte mitochondria-mediated apoptosis, accelerating the occurrence and development of atherosclerosis and CHD [42]. ROS can also act as second messengers, responding to the binding of extracellular signals to cell surface receptors through changes in their concentration. Through Ca^2+^ signal transduction, the mitogen-activated protein kinase signaling pathway, and the protein kinase B signaling pathway, ROS play a regulatory role in the process of cardiomyocyte signal transmission [43, 44]. Modern pharmacological studies have shown that various Chinese herbal extracts can treat CHD through their antioxidant effects.

#### 4.1.1. Ginsenosides

Ginsenosides are a group of nontoxic bioactive components with antioxidant effects produced by plants of the genus *Panax* [45, 46]. Ginsenosides Rb_1_, Rg_1_, and Rg_2_ have antioxidant effects, scavenge free radicals, improve myocardial ischemia and hypoxia, reduce intracellular calcium overload, and protect the myocardium [19, 47]. A large body of evidence suggests that ginsenosides Rb1, Rg1, and Rg2 possess protective abilities against CHD.

Ginsenoside Rb_1_ can reduce the occurrence of cardiomyocyte apoptosis, inhibit oxidative stress, inhibit the expression of apoptosis-promoting genes *Bax* and *Fas*, and upregulate mTOR signaling [48]. A recent study indicated that Rb1 could enhance the activity of antioxidant enzymes and reduce free radical-induced damage to the myocardium via activation of the PI3K/Akt/Nrf2 signaling pathway [49, 50].

Ginsenoside Rg2 could reduce oxidative stress injury and improve myocardial ischemia and hypoxia by regulating the activities of serum creatine kinase (CK), lactate dehydrogenase (LDH), lipid peroxides (LPOs), superoxide dismutase (SOD), and glutathione peroxidase (GPX) in rats [51]. Ginsenoside Rg2 was also shown to reduce oxidative stress in human epidermal keratinocytes [52].

Ginsenoside Rg1 may play a role in antioxidant defense by upregulating the AMPK/Nrf2/HO-1 signaling pathway. In addition, it exhibited protective effects against STZ-induced cardiac dysfunction [53]. Further studies indicated that Rg1 increased cell survival, promoted the expression of antioxidant proteins, and reduced ROS and apoptosis through the Nrf2/ARE signaling pathway [54]. Thus, it is suggested that Rg1 may be beneficial for the survival of cardiomyocytes via the inhibition of oxidative stress.

#### 4.1.2. Delphinidin-3-glucoside

Delphinidin-3-glucoside (DPg) is a bioflavonoid with strong antioxidant effects. DPg could decrease the expression of NOX2/NOX4 and caspase-3 induced by oxidized LDL (oxLDL), while reducing ROS production, p38 MAPK phosphorylation, NF-*κ*B p65 activity, and, importantly, the damage induced by oxidative stress [55].

Further, studies reported that DPg could induce autophagy through the AMPK/SIRT1 signaling pathway, thus protecting human umbilical vein endothelial cells (HUVECs) from oxLDL-induced oxidative stress [56].

#### 4.1.3. Total Flavonoids of Matsuba

Total flavonoids of matsuba are a natural extract from pine needles. It possesses antioxidant, anti-inflammatory, and antibacterial activities [57, 58]. The total flavonoids of matsuba can inhibit oxidative stress by upregulating the activity of SOD, GSH-PX, and CAT, while reducing malondialdehyde (MDA) content. In addition, the total flavonoids of matsuba can reduce the oxidative modification of LDL, directly capture and remove O_2_ and H_2_O_2_ radicals, and block the free radical-induced oxidative stress chain reaction, while also inhibiting the formation of toxic substances such as LPO and copolyludiene [17]. Therefore, the total flavonoids of matsuba may be a promising agent for treating CHD.

### 4.2. Ischemia-Reperfusion Injury

Ischemia-reperfusion injury refers to the increase in ROS and oxidative stress and the further damage of the mitochondrial ultrastructure function and metabolism due to the reoxygenation of tissue that has had its oxygen supply temporarily disrupted [59]. At present, it has become one of the decisive factors affecting the prognosis and survival of patients with CVD. The main contributors to the ROS increase are xanthine oxidase formation, neutrophil respiratory burst, mitochondrial single-electron reduction, catecholamine autoxidation, and intracellular Ca^2+^ overload [60]. The excessive production of ROS in the myocardial ischemic area can directly trigger myocardial cell apoptosis, inflammatory reactions, and energy metabolism disorders [61]. Several studies have shown that the active components of various CHMs can protect myocardial cells by regulating oxidative stress and have a good therapeutic effect on ischemia-reperfusion injury.

#### 4.2.1. Orientin

Orientin is a flavonoid component present in natural plant extracts. Orientin has been demonstrated to have antioxidant and anticancer properties, while also contributing to cardiac remodeling and the prevention of myocardial ischemia-reperfusion injury through enhanced antioxidant defense [62]. Studies have shown that orientin can inhibit the oxLDL-induced increase in TNF-*α*, IL-6, and IL-1*β*, as well as reducing levels of ROS [63]. Orientin also protects red blood cells from oxidative damage by reducing oxidative stress, increasing the activity of antioxidant enzymes, and maintaining the structural integrity of red blood cells [18]. Studies have also reported that orientin can regulate apoptosis via AMPK, Akt, mTOR, and Bcl-2 signaling and the maintenance of autophagic balance. In addition, orientin protects myocardial cells against hypoxia-reoxygenation injury [64]. Overall, orientin's protective effects are related to the inhibition of oxidative stress.

#### 4.2.2. Hawthorn Leaf Flavonoids

Hawthorn leaf flavonoids are the extract of the hawthorn dry leaves of *Rosaceae*. It possesses antioxidant, anti-inflammatory, and hypolipidemic activities [65]. Hawthorn leaf flavonoids were found to enhance the activity of antioxidant enzymes and inhibit the oxidative modification of LDL-C, while also improving oxidative stress-induced damage to the rat myocardium through the PKC-alpha signaling pathway. Further, the extract was shown to activate PPAR-*α* signaling to reduce blood triglycerides and regulate the vascular pathological response [66]. It was also found that hawthorn leaf flavonoids could protect vascular endothelial cells from free oxygen radicals by reducing lipid peroxidation and enhancing the activity of antioxidant enzymes and radical scavenging [67]. Therefore, hawthorn leaf flavonoids may be used for the prevention of myocardial injury induced by oxidative stress.

#### 4.2.3. Anemarrhenoside

Anemarrhenoside is a steroidal saponin monomer extracted from the dried rhizome of *Anemarrhena asphodeloides*, while also being its most abundant component [68–70]. Saponins E-I, E-II, B-II, B-III, and A-III of *Anemarrhena asphodeloides* can promote the production of SOD. Anemarrhenosides E-I and E-II can also inhibit the expression of prooxidative stress proteins and the abnormal aggregation of platelets [71]. It is reported that 35 different metabolites related to oxidative stress can be found in the H_2_O_2_-induced oxidative stress injury model of PC12 cells, and Anemarrhenoside B-II may play a protective role against oxidative stress by decreasing the formation of free radicals via regulation of oxidative stress-related metabolites [72]. In addition, Anemarrhenoside A-III has been reported to regulate the formation of ROS in cells and increase SOD and catalase (CAT) in a concentration-dependent manner, thus regulating intracellular oxidative homeostasis [73].

#### 4.2.4. Hesperidin

Hesperidin is a flavonoid widely found in lemon or citrus fruits and has strong antioxidant activity [74]. Hesperidin has been reported to have a wide range of pharmacological effects, such as regulation of lipid metabolism abnormalities, protection of cardiovascular endothelial cells, antioxidant activity, and regulation of autophagy [75, 76]. Research shows that hesperidin can inhibit oxidative stress by regulating Nrf2/ARE/HO-1 and TGF-beta1/Smad3 signal transduction [77]. The Nrf2/ARE-HO-1 axis mediated by ERS-PERK signaling is a new target for the treatment of myocardial ischemia-reperfusion injury [78]. Moreover, hesperidin can inhibit autophagy by activating the PI3K/Akt/mTOR signaling pathway, contributing to its myocardial protective effect on ischemia-reperfusion injury. Hesperidin's mechanism of action is related to the inhibition of oxidative stress [79].

### 4.3. Hypertension

Hypertension is a clinical syndrome characterized by high blood, which may be accompanied by functional damage of the heart, brain, and kidney [80, 81]. It has been proven that ROS play an important role in the pathophysiological process of hypertension. Increased oxidative stress is an important mediator of endothelial damage in hypertension, which is related to the increased synthesis of oxidants, such as hydrogen peroxide (H_2_O_2_) and nitric oxide (NO), and decreased antioxidant bioavailability [82, 83]. During hypertension, plasma myeloperoxidase levels and oxidative stress are significantly increased. Treatment with antioxidants inhibiting NADPH oxidase and ROS can effectively prevent the abnormal increase in blood pressure [84, 85].

ROS, as regulatory signaling molecules, can regulate the endothelial function of blood vessels as well as the relaxation and growth of vascular smooth muscle cells. In addition, ROS can stimulate cells to produce growth factor-like cell responses [86, 87]. MAPK signal transduction is a major mechanism that mediates vascular damage in hypertension. ROS-induced oxidative stress can inhibit the activity of tyrosine phosphatase and enhance the activity of MAPKs. Overexpression of MAPKs causes the abnormal activation of NF-*κ*B and HIF-1*α*, induces vascular damage caused by lipid peroxidation, and thus aggravates the vascular remodeling, which occurs during hypertension [88–90]. Recent studies have found that polyphenols in medicinal plants can slow down the progress of lipid peroxidation-induced vascular damage by regulating oxidative stress. These observations provide new options for the treatment of hypertension.

#### 4.3.1. Resveratrol

Resveratrol is a natural polyphenol present in peanuts, wine, mulberries, and other plants. It is an antioxidant compound that may be used to prevent and treat CVD [91–93]. Resveratrol can scavenge free oxygen radicals in the body [94], and studies have reported that it can prevent and treat hypertension by inhibiting oxidative stress [95, 96].

Resveratrol inhibits the formation of oxygen free radicals and reduces oxidative stress and blood pressure by enhancing the ability of redox proteins to alter the redox environment of cells [97]. Moreover, resveratrol can increase the expression of endothelial nitric oxide synthase (eNOS) by activating SIRT1 [98] and can effectively inhibit the uncoupling of eNOS and the generation of superoxide radicals through the inhibition of oxidative stress and ROS formation, thus maintaining normal vascular function and reducing blood pressure. The interaction between resveratrol and SIRT1 can also inhibit the expression of the angiotensin-II receptor, hindering vasoconstriction caused by angiotensin-II, resulting in blood vessel relaxation and a lower blood pressure [99].

#### 4.3.2. Tea Polyphenols

Tea is a traditional drink in China and one of the most popular drinks in the world. Tea polyphenols, the most important bioactive components of tea, also have beneficial effects for the prevention and treatment of hypertension [20]. Tea polyphenols can enhance endogenous SOD activity, inhibit lipid peroxidation, increase ATP levels, and inhibit the formation of free radicals [100].

Tea polyphenols have no regulatory effect on normal blood pressure but can significantly reduce the abnormal increases in blood pressure. The underlying mechanism is related to the increase in antioxidant enzyme activity and the decrease in oxidative stress [101]. It has been reported that intravenous injection of tea polyphenols can reduce blood pressure in rats with acute hypertension while also effectively inhibiting ROS formation, downregulating homocysteine-metabolizing enzymes and related metabolites in the rat aorta, and effectively reducing hypertension [102].

#### 4.3.3. Saponins of *Panax notoginseng*

Saponins of *Panax notoginseng* (SPN) have significant antioxidant effects on hypertension [103]. SPN can inhibit the formation of free oxygen radicals and regulated erythrocyte rheology in patients with hypertension. SPN can also significantly reduce MDA, increase the expression of SOD, increase the deformability, and reduce the aggregation of red blood cells [104]. *Panax notoginseng*, a medicinal plant, also contains ginsenoside Rb3, which can increase the endothelium-dependent relaxation of spontaneously hypertensive rats *in vitro*. The regulatory pathways behind this mechanism are related to antioxidant signaling [105]. SPN can also reduce the content of LPO in the brain and blood, allowing for enhanced resistance against aging and oxidative stress. Further, it increases the levels of GSH and CAT in serum, enhancing antioxidant defenses and lowering blood pressure [106]. Thus, SPN represents a novel option for the treatment of hypertension.

#### 4.3.4. Berberine

Berberine is a natural extract from *Rhizoma coptidis*. It is reported that berberine has therapeutic effects on a variety of CVDs. Further, berberine has antioxidant, anti-inflammatory, antiatherosclerosis, and antihypertensive effects [107–109].

The antihypertensive activity of berberine is due to inhibiting the activity of cholinesterase, thus activating the M-receptor on vascular endothelial cells and promoting the release of the vasodilator NO from endothelial cells, which results in peripheral vascular smooth muscle relaxation. This mechanism may also contribute to the antioxidant effect of berberine [110, 111]. In addition, it has been reported that berberine can regulate AMPK signaling and inhibit the overexpression of p-mTOR. Further, berberine can reduce the levels of CRP, TNF-*α*, and IL-6 in plasma. This extract is also able to reduce myocardial autophagy and apoptosis through the AMPK/mTOR pathway, thus alleviating myocardial injury [21]. In conclusion, berberine may be used to regulate blood pressure and prevent myocardial injury.

#### 4.3.5. Allicin

Allicin is a sulfur-containing compound extracted from the bulb of *Allium* (*Liliaceae*). Allicin is modified by alliinase and has strong hydrophobicity. It can quickly reach the intracellular space through the cell membrane [112, 113]. Allicin can exert an antioxidant effect by scavenging free radicals, reducing reactive oxygen species, inducing glutathione production, and regulating NOS. It has also been described as a potential drug for the prevention and treatment of hypertension [114, 115].

Studies have reported that allicin can strongly inhibit the formation of ROS, reduce H_2_O_2_-induced apoptosis, and increase SOD and NO levels, as well as the expression of eNOS. It is suggested that allicin protects vascular endothelial function through its antioxidant activity, thus reducing vascular endothelial damage caused by oxidative stress [116]. Studies have revealed that allicin can reduce the vascular response to angiotensin-II, downregulate the expression of AT1R/KEAP1, increase the expression of Nrf2, upregulate antioxidant enzymes, reduce oxidative stress, and relieve the high tension of blood vessels [114]. Therefore, allicin may be another promising agent for treating hypertension.

#### 4.3.6. Curcumin

Curcumin is a polyphenol compound extracted from the rhizome of a turmeric plant. Curcumin has anti-inflammatory, antioxidant, antifibrosis, and antitumor pharmacological activities [117–119]. Experimental studies have demonstrated curcumin's strong antioxidant effect. Curcumin inhibits oxidative stress by reducing the formation of peroxides in blood vessels, reduces vascular resistance, restores vascular reactivity, and inhibits the occurrence and development of hypertension [120]. Curcumin can also inhibit H/R-induced apoptosis and autophagy in H9c2 cardiomyocytes by upregulating Bcl-2 and inhibiting the expression of Bax, BECN1, BNIP3, and SIRT1 [22]. Curcumin regulates autophagy by inhibiting PI3K-AKT-mTOR signal transduction, promoting the dissociation of BECN1 and Bcl-2, preventing FOXO1 acetylation, and reducing oxidative stress, thus protecting vascular endothelial cell function and controlling blood pressure [121].

### 4.4. Heart Failure

Heart failure is considered the end-stage of various heart diseases, and cardiomyocyte apoptosis caused by oxidative stress has been described as the most important factor in heart failure [122]. Since the discovery of SOD in 1969, animal experiments and clinical trials have strongly supported the close relationship between oxidative stress and heart failure. Antioxidant drugs can prevent some of the pathological processes leading to heart failure, including cardiac hypertrophy, cardiomyocyte apoptosis, and ischemia-reperfusion injury [123]. Research by Liu et al. revealed that during the stage of compensatory cardiac hypertrophy, SOD and GSH-PX levels increased, LPO decreased, animal blood flow was stable, and the enhanced activity of the endogenous antioxidant system could effectively resist damage induced by exogenous ROS and reperfusion [124, 125]. It has been reported that in animal models of compensatory cardiac hypertrophy, the production of ROS by NADPH oxidases will gradually increase to a peak, which is at the level of heart failure decompensation [126].

Research has also reported that the increase in oxidative stress is related to the increase in autophagy during heart failure following pressure overload. In H9c2 cardiomyocytes, high concentrations of H_2_O_2_ increased autophagy. Therefore, the autophagy and oxidative stress may contribute to heart failure after chronic pressure overload [122]. Active components of CHM can regulate oxidative stress and autophagy and, thus, may be helpful in the treatment of heart failure [127, 128].

#### 4.4.1. Astragaloside IV

Astragaloside IV (As-IV) is a natural saponin purified from *Astragalus membranaceus*. As an exogenous antioxidant, As-IV can significantly protect myocardial cells and mitochondria during the process of heart failure [129–131]. This protective effect is mainly achieved by an increase in the reserve respiratory capacity of cardiomyocytes and mitochondrial ATP after oxidative stress injury, as well as through the increased activity of T-SOD, GSH-Px, and CAT in cardiomyocytes [132, 133]. As-IV can also improve the metabolic rate of cardiomyocytes, reduce the release of MDA and NOS, and inhibit the generation of free oxygen radicals, alleviating damage to the membrane of cardiomyocytes and thus improving their viability [134].

As-IV has also been reported to inhibit ROS and NADPH production by upregulating PGC-1*α* and TFAM, as well as to promote mitochondrial autophagy and mitochondrial biogenesis, contributing to the protection of damaged mitochondria through its antioxidant activity [135]. As-IV can also reduce the activities of CPK and LDH, reduce the loss of mitochondrial membrane potential, and ultimately slow down cardiomyocyte apoptosis [136–138].

#### 4.4.2. Tetramethylpyrazine

Tetramethylpyrazine (TMP) is the main active alkaloid ingredient of *Ligusticum* [139, 140]. TMP can reverse the PI3K/Akt signal pathway inactivation caused by hypoxia and reduce oxidative stress-induced cardiomyocyte apoptosis by downregulating miR-499a and upregulating SIRT1 signaling [141]. Oxidative stress induced by oxygen deficiency after heart failure causes great damage to cardiomyocytes, and TMP can directly enhance myocardial protection by reducing oxidative damage. Further, it can also inhibit cardiomyocyte apoptosis by regulating the expression of apoptosis-related proteins such as Bcl-2, Bax, and caspase-3 and the NF-*κ*B pathway [142].

Recent studies show that TMP could relieve vascular tension and counteract oxidative stress by scavenging ROS, downregulating ERK1/MAPK signaling, and inhibiting NF-*κ*B. TMP can also protect vascular endothelial cells from H_2_O_2_-induced injury by increasing the content of phosphatidylcholine, reducing the release of arachidonic acid, and inhibiting the phosphorylation of cytosolic phospholipase A [143, 144]. TMP, as an NADPH oxidase inhibitor and ROS scavenger, may be a potential antioxidant drug for the treatment of heart failure [145].

#### 4.4.3. Gastrodin

Gastrodin is a glucoside extracted from the rhizome of *Gastrodia elata* Blume [146]. It was found that gastrodin could inhibit the formation of and scavenge oxygen radicals as well as inhibiting LPO in the myocardium during the decompensated stage of heart failure [147, 148]. Further, gastrodin was found to inhibit oxidative stress by activating ERK1/2 signaling and reducing GSK-3*β* overexpression [149].

Gastrodin can also mitigate myocardial injury caused by myocardial ischemia-reperfusion and improve the morphology of damaged myocardial tissue. These effects were related to the enhancement of SOD-mediated inhibition of oxidative stress [150]. In addition, during myocardial ischemia-reperfusion, free oxygen radical production occurs along with the outflow of potassium ions, resulting in the overexpression of inflammatory cytokines and subsequent injury of myocardial cells. Reduced numbers of inflammatory and red blood cells in the interstitial space were observed in myocardial ischemia-reperfusion injury after gastrodin pretreatment. Further, the degree of myocardial cell damage was also lower, which may be related to the antioxidant mechanism of gastrodin [151].

#### 4.4.4. Safflower

Safflower is an extract of *Crocus sativus* L. and is commonly used in the treatment of CVD. Clinical studies have shown that safflower has antioxidant and antiarrhythmic effects, as well as protective effects on damaged myocardium [152–154]. Thus, safflower is an antioxidant with potential for the prevention and treatment of heart failure. It has been reported that safflower can significantly inhibit the overexpression of proapoptotic genes *Bad* and *Bax* by inducing autophagy. Further, safflower treatment reversed apoptosis induced by angiotensin-II (Ang-II) in H9c2 cells [155]. Safflower also inhibited H_2_O_2_-induced oxidative stress injury by upregulating Nrf2/HO-1/NADPH/NQO1 signaling and Akt phosphorylation [156]. Therefore, safflower may have potential for the treatment and prevention of heart failure through its antioxidant and antiapoptotic activities.

#### 4.4.5. Ferulic Acid

Ferulic acid (FA) is a phenolic acid found in *Angelica sinensis*, chuanxiong, and other medicinal plants. FA has strong antioxidant capacity and not only inhibits free radical production but also downregulates free radical activity [157]. It has been found that FA can reduce the myocardial infarction area and LDH, CK, and cardiac troponin levels in a dose-dependent manner. FA protects myocardial tissue by activating PI3K/Akt/mTOR signaling and restoring autophagic flux, as well as through its antioxidant activity [158]. It can also increase the expression of Beclin-1/LC3-II and ATG5, while protecting cardiomyocytes from caspase-dependent and caspase-independent apoptosis by activating HSP70 and Bcl-2 [159]. At the same time, FA can counteract excessive ROS production and induce autophagy, thus inhibiting cell apoptosis [160]. Therefore, the antiapoptotic effect of FA may be mediated by its antioxidant and autophagy-inducing activities.

### 4.5. Arrhythmia

Arrhythmia is caused by abnormal sinoatrial node activity. Studies have shown that the mechanism of arrhythmia is closely related to oxidative stress [161]. Slow activation of the sinus node caused by oxidative stress or abnormal conduction can lead to arrhythmia [162]. Studies have shown that ROS, MDA, and other oxidative stress markers in the serum of patients with tachyarrhythmia were significantly increased, while the expression of SOD, TAC, GSH, and other antioxidant markers was decreased. In addition, oxidative stress can promote the occurrence of atrial fibrillation, a vicious circle that will eventually lead to the aggravation of arrhythmia symptoms [163]. These observations suggest that oxidative stress plays an important role in the pathogenesis of arrhythmia. At present, traditional antioxidants cannot achieve the desired therapeutic effect. However, active components derived from medicinal plants can regulate the heart rate by inhibiting oxidative stress, suggestive of their potential as an antiarrhythmic drug in the future.

#### 4.5.1. Paeonol

Paeonol is an extract of the rhizome of *Paeonia suffruticosa*, which has antibacterial, anti-inflammatory, and antioxidant effects [164, 165]. Paeonol can prevent the occurrence of arrhythmia, shorten the duration of atrial fibrillation or the conduction block, and protect against ischemia-reperfusion myocardial injury. It has been reported that ischemia-reperfusion causes an increase in free radicals, a decrease in SOD activity, and an increase in MDA content, in parallel to accelerated myocardial injury and increased instability of cardiac bioelectrical conduction, leading to arrhythmia. However, paeonol can enhance SOD scavenging of endogenous radicals and reduce LPO levels, potentially exerting antiarrhythmic effects and improving myocardial injury. Paeonol can also block calcium channels of cardiomyocytes, inhibit the transient outward potassium current, selectively block the fast sodium channel, reduce the range of phase 0 depolarization, and shorten the time course of action potential in ventricular muscle [166]. These outcomes may be related to paeonol's ability to inhibit oxidative stress.

#### 4.5.2. Matrine

Matrine is a natural extract from *Sophora flavescens*. It possesses antioxidant, antiviral, and antiarrhythmic activities [167–169]. Matrine can directly inhibit the flow of sodium ions outside the myocardial cell membrane and maintain the normal heart rhythm [170]. It can also reduce cardiomyocyte stress and improve ectopic heartbeats by affecting the potassium and sodium ion transfer system at the cardiomyocyte membrane [171]. Research has shown that matrine can prolong the refractory period of the atrium and ventricle by inhibiting oxidative stress, can reduce the excitability of the atrium and the ventricular muscle, and can pace the conduction system. Further, matrine stabilizes the heart rhythm by inhibiting oxidative stress and increasing endogenous antioxidant activity, thus protecting the structure and function of mitochondria in cardiomyocytes [172, 173].

### 4.6. Acute Myocardial Infarction

Acute myocardial infarction (AMI) is a disease with high mortality and is caused by persistent ischemia and hypoxia of the coronary artery. Early reperfusion following myocardial infarction is the most essential form of treatment. However, when blood supply is restored, excessive free oxygen radicals will damage tissues leading to ischemia-reperfusion injury. Studies have shown that myocardial ischemia-reperfusion injury is closely related to oxidative stress and myocardial autophagy. Autophagy can protect cardiomyocytes from ischemia and accelerate cell death during reperfusion [174, 175]. Moreover, trimetazidine treatment can reduce the oxidative stress and autophagic flux induced by acute myocardial infarction, thus reducing infarction size. Studies have indicated that cardiomyocytes, oxidative stress, and autophagy are involved in the pathological process of AMI and ischemia-reperfusion injury. In AMI, the increase in ROS/autophagy and the decrease in SOD can enhance oxidative stress and aggravate myocardial injury. The induction of autophagy may be related to the activation of the ROS-ATM-LKB1-AMPK signal axis [176]. This axis could represent a new therapeutic target in the treatment of AMI through CHM active components.

#### 4.6.1. Astragalus Polysaccharides

Astragalus polysaccharides (APS) are water-soluble polysaccharides with biological activity, extracted from *Astragalus*. APS are increasingly considered potential exogenous antioxidants. It is reported that APS have antioxidant, antiviral, and anti-inflammatory pharmacological effects [177, 178]. APS can remove superoxide anions and hydrogen free radicals, improve the activity of SOD, GPX, and CAT, and reduce the levels of LPO. APS can also reduce cell apoptosis, the production of DHE, cytosolic nitrotyrosine products, and nuclear oxidative stress (8-OH-AD), while reducing ROS generation [179]. APS can also reduce the troponin and creatine phosphokinase, as well as the mRNA expression of Bcl-2, Bax, caspase-3, p53, Apaf-1, and AIF, leading to an improved antioxidant capacity and enhanced protection against cardiac injury caused by myocardial cell apoptosis [180].

#### 4.6.2. Quercetin

Quercetin is one of the most well-known flavonoids. It can form complexes with superoxide anions (O_2_^−^) to reduce the production of oxygen radicals and couples with Fe_2_^+^ to prevent the formation of Fenton radicals. Quercetin also reduces the consumption of NADPH by inhibiting aldose reductase, improving the body's antioxidant capacity [181]. Quercetin can also scavenge free radicals produced in macrophages, inhibit the oxidation of LDL, protect tocopherol, and regenerate oxidized *α*-tocopherol [182].

According to a previous report, quercetin can maintain proper ST segment elevation in myocardial infarction model rats, reduce the level of LPO products in the rat serum and heart, and protect the damaged myocardium [183]. Quercetin could reverse the increase in NO, MDA, MPO, and caspase-3 activity, while decreasing GSH and SOD activity in the ischemia-reperfusion group. It has also been suggested that quercetin can alleviate tissue injury induced by AMI through its antioxidant and antiapoptotic effects [184]. Moreover, quercetin protects vascular endothelial cells and reduces blood pressure through antioxidant activity [185]. Thus, quercetin is a potential drug for the treatment of AMI and hypertension in the future.

#### 4.6.3. Tanshinone II-A

Tanshinone II-A is a lipid-soluble phenanthraquinone extracted from the rhizome of *Salvia miltiorrhiza*. It possesses antioxidant properties, regulates autophagy, and has certain advantages in the protection of myocardial cells [186, 187]. Tanshinone II-A can alleviate oxidative stress injury of H9c2 cells induced by DOX, enhance autophagy in H9c2 cells, and mitigate myocardial injury [188]. Tanshinone II-A exerts its antioxidant effects through NRF-2, reducing DOX cardiotoxicity. These antioxidant properties of tanshinone II-A play an important role in protecting myocardial cells after AMI [189]. Autophagy is a protective mechanism allowing cells within plaques to fight against and resist oxidative stress. An excessive reduction or increase in autophagic activity will affect the extent of oxidative stress-induced damage and atherosclerotic plaque development. Tanshinone II-A can also affect autophagic activity by regulating autophagy genes ATG and LC3, contributing to its antioxidant effects [190].

#### 4.6.4. Gypenoside

Gypenoside (GPS) is a commonly used drug for the prevention and treatment of CVDs [191]. Its benefits include antioxidant and antiatherosclerosis properties, as well as protection of the damaged myocardium. GPS can enhance the antioxidant capacity of aging rats by increasing SOD activity and can promote c-sis gene expression in endothelial cells, as well as the synthesis and release of NO, while also improving blood circulation of the coronary artery. GPS has also been reported to restore the normal redox state of ox-LDL in HUVECs through antioxidant regulation via PI3K/Akt. Further, GPS upregulated the ratio of Bcl-2 to Bax and inhibited the expression of caspase-3, leading to apoptosis [192]. GPS has protective effects on myocardial ischemia and systolic function in rats and exerts its cardiotonic and central inhibitory effects by inhibiting the activities of Na^+^/K^+^-ATPase in the heart [193]. GPS contributes to the resistance of oxygen radical damage to the heart, protects the integrity of the myocardial cell membrane, and supports normal diastolic function of the heart during acute myocardial ischemia [194].

#### 4.6.5. Soybean Isoflavones

Soybean isoflavone (SI) is a bioactive secondary metabolite formed during soybean growth. Studies have shown that SI can protect the cardiovascular system through its antioxidant effects [195–197]. SI enhanced the activity of SOD, decreased the level of thiobarbituric acid reactant in plasma, and enhanced the antioxidant capacity of plasma, as well as the activity of GSH-PX in erythrocytes [198]. Moreover, SI inhibited the production of peroxides and the activity of NADPH oxidase in ischemia injury [199]. Studies have reported that different concentrations of SI can inhibit the apoptosis of vascular endothelial cells in a concentration-dependent manner, thus reducing vascular endothelial damage [200].

#### 4.6.6. Hydroxy Safflower Yellow

Hydroxy safflower yellow (HSYA) is a natural active component of *Carthamus tinctorius* L. of *Compositae*, which has significant anticoagulant and antioxidant activity. HSYA reduces the level of LDH and caspase-3 in the heart of ischemia-reperfusion injury models, indicative of lower apoptosis rates [201]. HSYA can also inhibit the apoptosis of myocardial cells after AMI by increasing the level of Bcl-2/Bax [202], improve the dysfunction of mitochondrial energy metabolism by activating the PI3K/Akt signaling pathway, and play a protective role for the myocardium [203]. HSYA is a water-soluble antioxidant active component, capable of clearing oxygen radicals and inhibiting LPO generation, thus protecting the myocardial cell membrane. It has also been found that HSYA has a chalcone structure and various phenolic hydroxyl groups. HSYA's antioxidant effect may be related to the action of these phenolic hydroxyl groups [204]. HSYA can also increase the expression of NADPH and NQO1, increase the phosphorylation of Akt, and inhibit H_2_O_2_-induced oxidative stress injury by activating Nrf2/HO-1 [156]. Overall, there is a growing body of evidence for the antioxidant properties of HSYA.

## 5. Discussion

In the current review, the effects and mechanisms of action of the active components of 27 natural extracts in various CVDs were discussed with regard to oxidative stress. Previous studies found that oxidative stress regulation by these active components is achieved through a variety of pathways. Evidence shows that these active ingredients could be used for therapy in the future. However, further experimental studies are needed to elucidate the exact molecular mechanisms of components before their implementation in clinical use. Overall, compounds derived from medicinal plants work through various mechanisms to counteract oxidative stress.

The therapeutic efficacy of natural drugs and their active ingredients has been reported not only in cell and animal models but also in the clinic. Recent clinical studies have found that SI has therapeutic effects on heart failure caused by ischemic cardiomyopathy (IC). SI was shown to increase the expression levels of Nrf2 and SOD and to reduce the expression of C-reactive protein, 8-isopropanol, MDA, IL-6, and TNF-*α* in patients with heart failure, improving the antioxidant capacity of patients with IC by upregulating Nrf2 and treating heart failure [205].

Chekalina et al., through a clinical study of 85 patients with coronary heart disease, found that quercetin can adjust the central hemodynamic parameters of patients with stable coronary heart disease and can improve myocardial ischemia [206]. The results of previous clinical studies have shown an improvement in left ventricular systolic function and left ventricular ejection fraction (EF) of patients, after two months of quercetin treatment. These clinical studies suggest that quercetin had cardioprotective effects in patients with coronary heart disease.

Clinical studies have also found that resveratrol had therapeutic effects on central hemodynamic parameters and myocardial ischemia in patients with stable coronary heart disease. The studies showed that the left ventricular systolic and diastolic function and the left ventricular ejection fraction (EF) were improved in patients with coronary heart disease, after resveratrol treatment. The phase ratio of conduction blood flow *E*/*A* also improved, and it was dominant in the research group. After resveratrol treatment, the DT value decreased significantly along with the number of PACs and PVCs. This research suggested that resveratrol has a significant protective effect on the heart in clinical settings [207].

Recent studies have also shown that the active ingredients of natural drugs combined with other drugs can result in synergistic therapeutic effects. A randomized controlled trial (RCT) found that tanshinone II-A sodium sulfonate injection (STS) can be used to treat CHD. The results of the study showed that the adjuvant treatment with STS significantly reduced the incidence of cardiac shock, heart failure, and arrhythmia, with no serious adverse events related to STS. STS combined with conventional medication is more effective than conventional medication alone, with fewer side effects [208].

Zhang et al. found that trimetazidine combined with berberine can have a therapeutic effect on endothelial function in patients with CHD and essential hypertension (CCP) [209]. eNOS mRNA expression (*P* < 0.05), NO level (50.75 ± 2.75 mol/l) (*P* < 0.05), and FMD value (14.02 ± 2.39) were significantly higher after the combination treatment than before the treatment (*P* < 0.05). The results suggested that the combination of trimetazidine and berberine increased blood NO content, promoted the endothelium-dependent relaxation function of the brachial artery, and helped treat CCP.

In summary, since the multilevel, multichannel, and multitarget action of natural medicines can effectively reduce the side effects of single-chain action, there is great potential for clinical efficacy. However, most of the research on the targeted treatment of cardiovascular diseases using natural medicines and active ingredients has been conducted in cell and animal experiments, and only a few clinical studies exist. A better understanding of the pathology of cardiovascular diseases and the pharmacological effects of natural drugs and active ingredients could aid future large-scale clinical research studies on these drugs and their targets.

Recently, the clinical application of Chinese medicine has attracted considerable attention. Traditional Chinese medicine contains a variety of active ingredients with antioxidant effects. Moreover, the active ingredients show network synergy or antagonistic effects. The clinical compatibility and application of traditional Chinese medicine follow the traditional Chinese medicine theories, and various active ingredients contained in different traditional Chinese medicines have different degrees of synergy and antagonism [210]. The interaction among several active ingredients will affect the absorption, metabolism, efficacy, and toxicity of other active ingredients. For example, the compound Huangdai is used to treat granulocytic leukemia. The main ingredients of the prescription are tetraarsenic tetrasulfide, indirubin, and tanshinone II-A. Studies have found that the combination of these three drugs can synergistically enhance anticancer activity compared with each drug alone or in combination of two drugs [211]. Danshen dripping pills and Shensongyangxin capsules commonly used in the clinical treatment of cardiovascular diseases contain Chinese herbal medicines such as ginseng, Danshen, *Panax notoginseng*, and *Ophiopogon japonicus*. The active ingredients of these drugs are ginsenosides, tanshinones, and total saponins of notoginseng, each of which can regulate oxidative stress. When combined, these active ingredients work together to produce a synergistic effect [212, 213].

Chinese medicine is complex, with diverse chemical components affecting organisms through various biological reactions. This diversity determines whether the effects of the different active ingredients in Chinese medicines will be synergistic, additive, and antagonistic. However, it is yet unclear which combinations of active ingredients have synergistic effects and antagonistic effects or which combinations may increase toxicity. At the effect level, avoid invalid and negative action modes, and which active ingredients will strengthen the primary pharmacological effect and which will negate it, as well as effective/optimal doses of each active ingredient in the combinations, need to be determined. The similarities and differences between the antioxidant mechanisms of medicinal plants and the mechanisms by which they exert therapeutic effects on the human cardiovascular system are also worth discussing. One of the advantages of medicinal plants is the synergistic action of multiple plant chemical components. Williamson first proposed the synergistic effect of natural extracts in 2001 [214]. Synergistic effect refers to multiple pathways being simultaneously targeted and engaged. There are various pathways and targets involved in oxidative stress signaling, and natural drugs or active ingredients often act on multiple pathways and targets for the effective treatment of the disease. “Synergy multitarget” and “antagonistic multitarget” may explain the effects observed from the combination of the active ingredients in traditional Chinese medicine.

In order to elucidate the mechanism of action of traditional Chinese medicine, the pathways and targets acted upon by each active ingredient alone and in different combinations need to be studied. It is also necessary to find a suitable entry point and establish a reasonable pharmacological model based on multiple omics researches such as genomics, proteomics, and metabolomics. Determining the mechanisms of action of the active ingredients can not only explain how the various ingredients in Chinese medicines function alone or in combination but more importantly may lead to the discovery of new mechanisms of action and synergistic effects of the active ingredients and lay a foundation for the innovation of Chinese medicine and the development of Chinese medicine theory.

## 6. Conclusions

Medicinal plants are used to treat cardiovascular disease through their antioxidant properties, and remarkable effects have been reported. The current review summarized the mechanisms of active plant components in the treatment of CHD, hypertension, heart failure, ischemia-reperfusion, and arrhythmia. The dosing and timing of active component administration require further study. Future research should also investigate the synergistic effects of multiple bioactive plant components. In addition, large-scale clinical studies should be conducted to confirm the clinical effectiveness and safety of natural medicines and their effective active ingredients.

## Figures and Tables

**Table 1:** Detailed information on bioactive ingredients targeting oxidative stress.

No.	Active ingredients	Natural drug	Mechanism of action	Treatment disease
1.	Ginsenoside Rb_1_	*Panax ginseng*	(1) Inhibits the expression of proapoptotic genes *Bax*, *Bad*, and *Fas* (2) Increases the activity of antioxidant enzyme (3) Reduces the oxygen free radicals	(1) Coronary heart disease (2) Ischemia-reperfusion injury
2.	Ginsenoside Rg_1_	*Panax ginseng*	(1) Inhibition of *caspase-3*, *Bax*, and *ap-JNK* expression (2) Increases the expression of *p-ERK* (3) Reduces ROS	(1) Coronary heart disease (2) Ischemia-reperfusion injury
3.	Ginsenoside Rg_2_	*Panax ginseng*	(1) Inhibition of *CK* and *LDH* (2) Reduced LPO (3) Increases the activity of *SOD*, *CAT*, and *GSH-Px*	(1) Coronary heart disease (2) Ischemia-reperfusion injury
4.	Delphinidin-3-glucoside	Anthocyanidin	(1) Inhibits the expression of *NOX2/NOX4* and *caspase-3* (2) Reduces ROS (3) Induces autophagy through *AMPK/SIRT1*	(1) Coronary heart disease (2) Ischemia-reperfusion injury
4.	Total flavonoids of matsuba	Matsuba	(1) Reduce O_2_ and H_2_O_2_ (2) Inhibit the formation of LPO and MDA (3) Increase the activity of *SOD*, *GSH-Px*, and *CAT*	(1) Coronary heart disease (2) Atherosclerosis (3) Hyperlipidemia
5.	Orientin	*Passiflora* leaves	(1) Reduces ROS (2) Increases the activity of antioxidant enzyme (3) Regulating *AMPK*, *Akt*, *mTOR*, and *Bcl-2*	(1) Coronary heart disease (2) Atherosclerosis
6.	Hawthorn leaf flavonoids	Genus	(1) Inhibit the formation of LPO (2) Increase the activity of antioxidant enzyme (3) Inhibit free radical reaction	(1) Coronary heart disease (2) Atherosclerosis (3) Hyperlipidemia
7.	Anemarrhenoside	*Anemarrhena asphodeloides*	(1) Promoting the production of *SOD* (2) Inhibit the expression of prooxidative stress protein (3) Reduce ROS	Ischemia-reperfusion
8.	Hesperidin	Citrus fruits	(1) Regulating *Nrf2/ARE/HO-1* and *TGF-beta1/Smad3* signal transduction (2) Regulating *PI3K/Akt/mTOR* signaling pathway	Ischemia-reperfusion
9.	Resveratrol	Peanuts, red wine, mulberries, etc.	(1) Inhibits the formation of oxygen free radicals (2) Reduces ROS (3) Increases the expression of eNOS by activating *SIRT1*	(1) Hypertension (2) Ischemia-reperfusion
10.	Tea polyphenols	Tea	(1) Increase the activity of *SOD* (2) Inhibit the formation of LPO (3) Downregulating *Hcy* metabolic enzymes	(1) Hypertension (2) Ischemia-reperfusion
11.	Saponins of *Panax notoginseng*	*Panax notoginseng*	(1) Inhibit the formation of LPO and MDA (2) Increase the activity of *SOD* (3) Upregulate the expression of *GSH* and *CAT*	Hypertension
12.	Berberine	*Rhizoma coptidis*	Regulates the *AMPK/mTOR* signaling pathway	Hypertension
13.	Allicin	*Allium* in *Liliaceae*	(1) Scavenging free radicals (2) Inhibits the formation of ROS (3) Increases the activity of antioxidant enzyme	Hypertension
14.	Curcumin	Rhizome of a turmeric plant	(1) Reducing the formation of peroxides (2) Inhibiting the expression of *Bax*, *beclin-1*, *BNIP3*, and *SIRT1* (3) Inhibiting *PI3K-AKT*-*mTOR* signal transduction	Hypertension
15.	Astragaloside IV	*Astragalus propinquus*	(1) Increases the activity of *T-SOD*, *GSH-PX*, and *CAT* (2) Inhibits the formation of MPO, *NADPH*, MDA, and ROS (3) Inhibits the activity of *CPK* and *LDH*	(1) Heart failure (2) Heart remodeling (3) Ventricular dysfunction
16.	Tetramethylpyrazine	*Ligusticum chuanxiong*	(1) Increased the expression level of *microRNA-499a* (2) Upregulation of sirtuin1	(1) Heart failure (2) Coronary heart disease
17.	Gastrodin	*Gastrodia elata*	(1) Inhibits the formation of LPO and oxygen free radicals (2) Increases the activity of *SOD*	Heart failure
18.	Safflower	*Crocus sativus L.*	(1) Inducing autophagy (2) Increasing the expression of *Nrf2/HO-1/NADPH/NQO1*	Heart failure
19.	Ferulic acid	*Angelica sinensis*	(1) Inhibition of *CK* and *LDH* (2) Activating the *PI3K*/*Akt*/*mTOR* signaling pathway (3) Reduces ROS	Heart failure
20.	Paeonol	*Paeonia suffruticosa*	(1) Increases the activity of *SOD* (2) Inhibits the formation of LPO and oxygen free radical	(1) Arrhythmia (2) Coronary heart disease
21.	Matrine	*Sophora flavescens*	Increases the activity of antioxidant enzyme	Arrhythmia
22.	Astragalus polysaccharide	*Astragalus propinquus*	(1) Increases the activity of *SOD* (2) Inhibits the formation of LPO, ROS, and oxygen free radical (3) Increased the expression of *8-OH-AD*	(1) Coronary heart disease (2) Acute myocardial infarction
23.	Quercetin	*Dendrobium nobile*	(1) Inhibits the expression of aldose reductase (2) Inhibits the formation of MPO and *NADPH* (3) Inhibits the oxidation of low-density lipoprotein (LDL)	(1) Acute myocardial infarction (2) Ischemia-reperfusion
24.	Tanshinone II-A	*Salvia miltiorrhiza*	(1) Upregulation of *Nrf-2* (2) Regulates the autophagy genes *ATG* and *LC3*	(1) Coronary heart disease (2) Acute myocardial infarction
25.	Gypenoside	*Gynostemma pentaphyllum*	(1) Increases the activity of *SOD* (2) Inhibits the formation of oxygen free radical	Acute myocardial infarction
26.	Soybean isoflavone	*Glycine max*	(1) Increases the activity of *SOD* and *GSH-Px* (2) Inhibits the activity of *NADPH* and *NOX*	(1) Acute myocardial infarction (2) Hyperlipidemia
27.	Hydroxy safflower yellow	*Carthamus tinctorius* L.	(1) Activating the *PI3K/Akt* signaling pathway (2) Increases the expression of *NADPH* and *NQO1*	Acute myocardial infarction

ROS: reactive oxygen species; ap-JNK: C-Jun N-terminal kinase- (JNK-) c-Jun/activated protein (AP); p-ERK: protein kinase R- (PKR-) like endoplasmic reticulum kinase; CK: creatine kinase; LDH: lactate dehydrogenase; LPO: lipid peroxidation; SOD: superoxide dismutase; CAT: catalase; MDA: malondialdehyde; GSH-Px: glutathione peroxidase; eNOS: endothelial nitric oxide synthase; NADPH: nicotinamide adenine dinucleotide phosphate; NOX2: NADPH oxidase 2; NOX4: NADPH oxidase 4; AMPK: adenosine 5′-monophosphate- (AMP-) activated protein kinase; SIRT1: sirtuin1; PI3K: phosphatidylinositol 3-kinase; Akt: serine/threonine kinase Akt; mTOR: mammalian target of rapamycin; Nrf2: nuclear factor erythroid 2-related factor 2; ARE: antioxidant response element; HO-1: heme oxygenase 1; TGF-beta1: transforming growth factor beta 1; NQO1: NAD(P)H quinone dehydrogenase 1.
